# Supplementary material for: Genomic signatures of heterokaryosis in the oomycete pathogen Bremia lactucae
Source: Nat Commun. 2019 Jun 14;10:2645. doi: 10.1038/s41467-019-10550-0 (PMC6570648; doi:10.1038/s41467-019-10550-0)
Supplement: Supplementary file 1 — Supplementary Information [file 41467_2019_10550_MOESM1_ESM.pdf]

Supplementary Information

Genomic signatures of heterokaryosis in the oomycete pathogen *Bremia lactucae*

Fletcher *et al.*

Contents:

Supplementary Figures:

Supplementary Figure 1. Long tandem repeat (LTR) nucleotide divergence.

Supplementary Figure 2. Heat map showing the distribution of orthogroups where a Pfam domain, found to be depleted in downy mildews, is detected as encoded by at least one gene model of that orthogroup.

Supplementary Figure 3. Sequence coverage of different groups of genes in the *B. lactucae* assembly.

Supplementary Figure 4. Bar plots of alternative allele frequencies for each SNP detected between the tested isolate and the SF5 assembly.

Supplementary Figure 5. Bar plots of alternative allele frequencies for each SNP detected between 4 progeny isolates and the SF5 assembly.

Supplementary Figure 6. Bar plots of alternative allele frequencies for each SNP detected between 11 C82P24 single spore derivatives and the SF5 assembly.

Supplementary Figure 7. Bar plots of alternative allele frequencies for each SNP detected between nine C98O622b single spore derivatives and the SF5 assembly.

Supplementary Figure 8. Bar plots of alternative allele frequencies for each SNP detected between heterokaryons generated *in silico* and the SF5 assembly.

Supplementary Figure 9. Bar plots of alternative allele frequencies for SNPs unique to each homokaryotic nuclear type in the heterokaryotic derivatives and the SF5 assembly.

Supplementary Figure 10a. Assembly workflow used to achieve the final *B. lactucae* SF5 assembly.

Supplementary Figure 10b. Genome assembly checkpoints.

Supplementary Table:

Supplementary Table 1. Total nuclear DNA content of 39 *Bremia lactucae* isolates, including SF5. Sexual progeny isolates generated in the lab are italicized. Table is sorted by mean nuclear content (Mb).

Supplementary Table 2. Comparative statistics of downy mildew genome assemblies and select *Phytophthora* assemblies.

Supplementary Table 3. Annotation statistics of 25 oomycete genome assemblies in the public domain, plus calculated mean and standard deviation.

Supplementary Table 4. Orthology of previously reported candidate effectors of *B. lactucae* to proteins annotated in the SF5 genome.

Supplementary Table 5. Pathogenicity domains annotated in genome assemblies of Peronosporales spp.

Supplementary Table 6. Nuclear DNA content of 12 asexual single sporangia derivatives of *B. lactucae* isolate C82P24.

Supplementary Table 7. Collection dates and geographic regions of *B. lactucae* diversity panel isolates sequenced in this study.

Supplementary Table 8. Sequencing statistics for *B. lactucae* isolates, including number of reads sequenced, percent that map to the SF5 assembly, coverage and under which BioProject they may be found.

Supplementary Table 9. SRA accession numbers for oomycete sequences downloaded for this study.

Supplementary References.

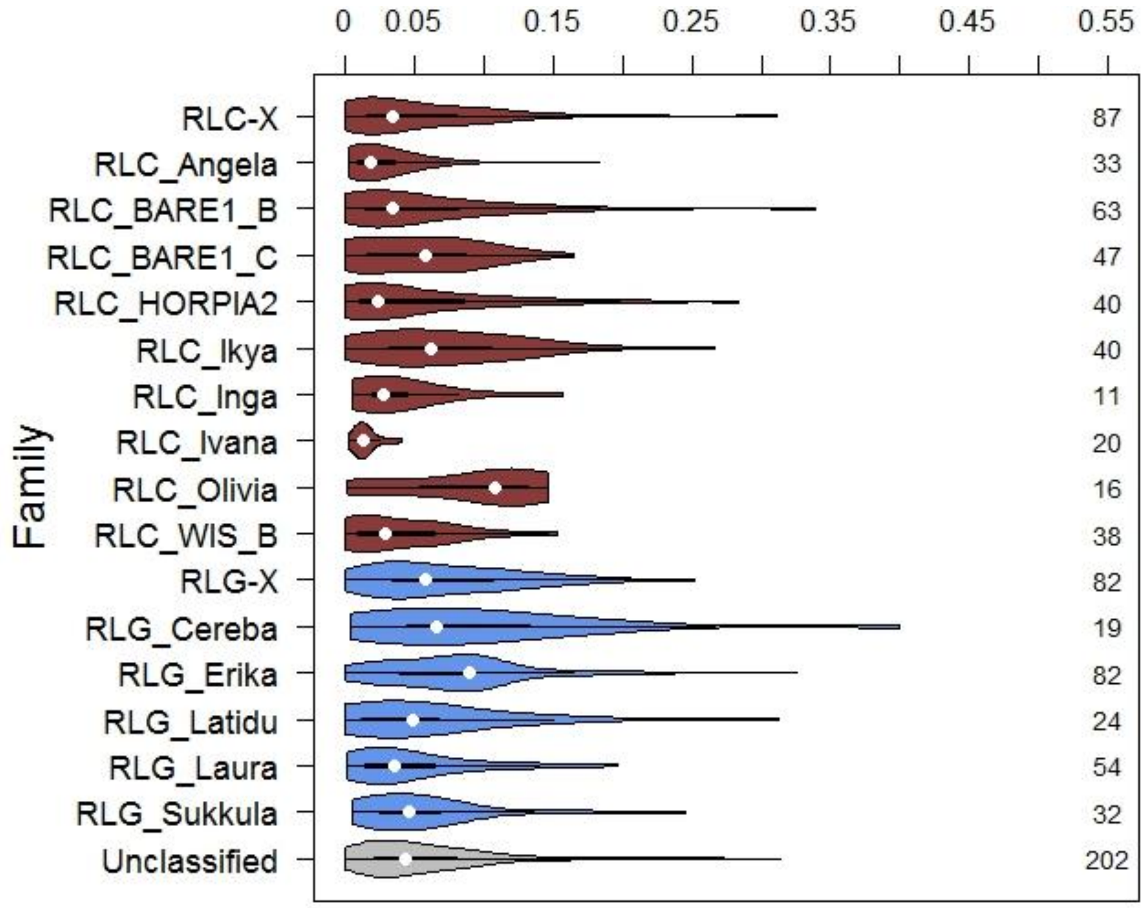

Supplementary Figure 1. Long tandem repeat (LTR) nucleotide divergence.

Estimates of divergence between LTRs identified in the *B. lactucae* assembly. LTRs are identical upon insertion of an LTR retrotransposon. Their divergence can therefore be used to infer the timing of insertion. RLC (red) = LTR *Copia* elements. RLG (blue) = LTR *Gypsy* elements.

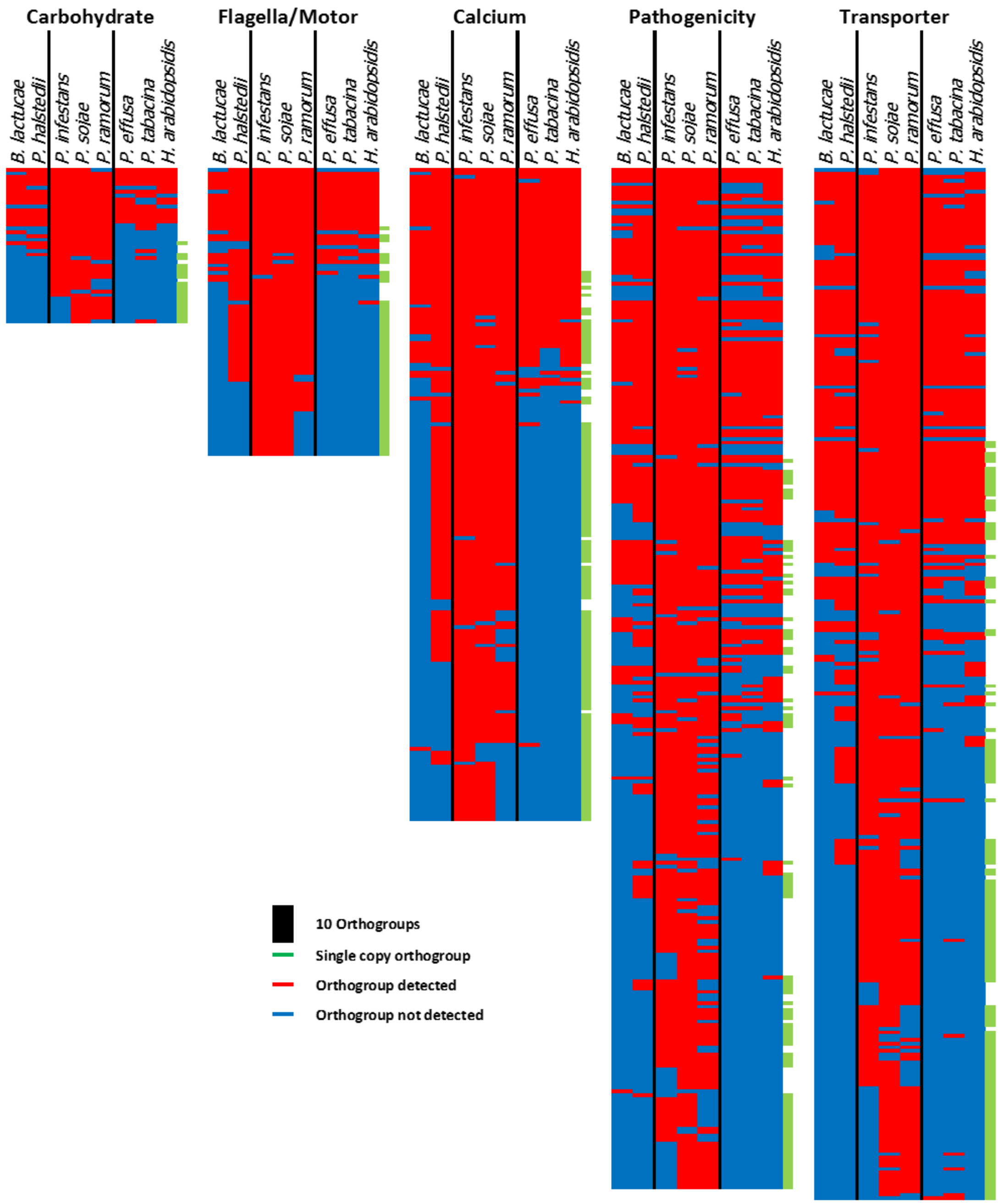

Supplementary Figure 2. Heat map showing the distribution of orthogroups where a Pfam domain, found to be depleted in downy mildews, is detected as encoded by at least one gene model of that orthogroup.

Red indicates the orthogroup contains at least one model from the species, blue indicates that no model is detected from the species. Green tabs on the right of the row indicates that the orthogroup contains a maximum of one gene from each species except for *P. effusa* and *P. tabacina* where two models were permitted because two isolates were combined in the analysis.

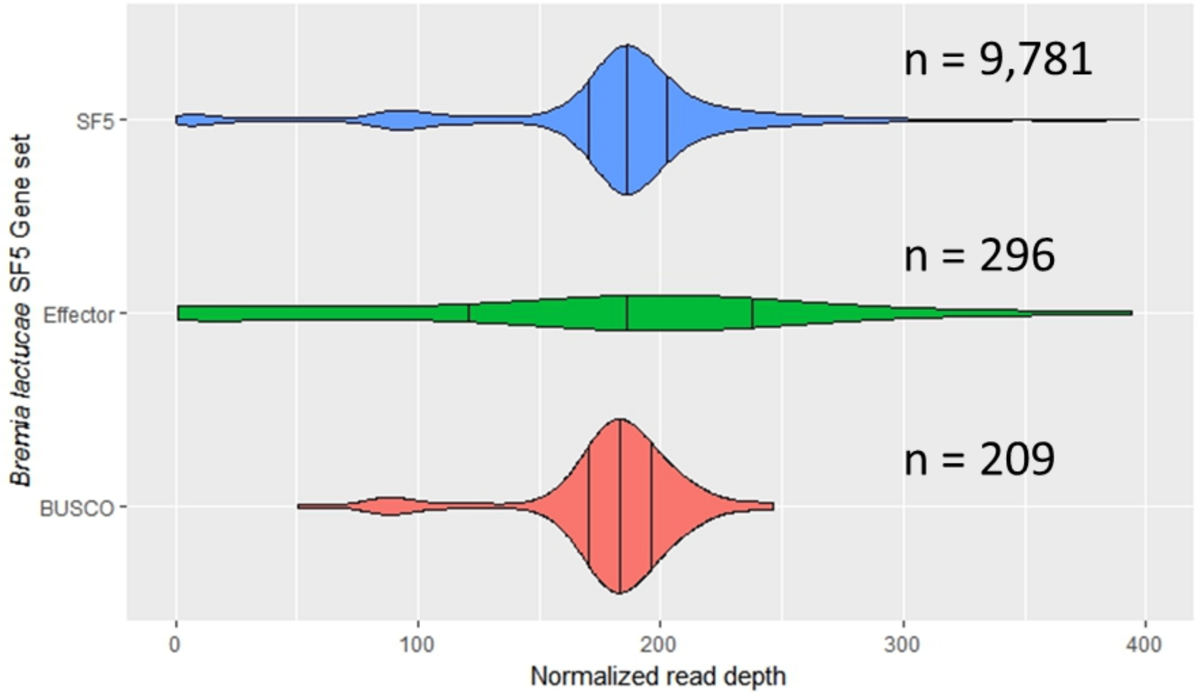

Supplementary Figure 3. Sequence coverage of different groups of genes in the *B. lactucae* assembly.

The majority of annotated genes have coverage at approximately 180x, equal to the sequencing depth. This indicates that the majority of genes are assembled as single consensus sequences. A minority of genes have coverage at approximately 90x. This is consistent with alleles being assembled independently or hemizygous regions. The same pattern is observed in the BUSCO genes, of which eight were detected as being duplicated. The effector portion of the genomes does not display the same distribution. This may be due to effector alleles being assembled independently, or alternatively a high rate of divergence between haplotypes resulting in poor rates of mapping.

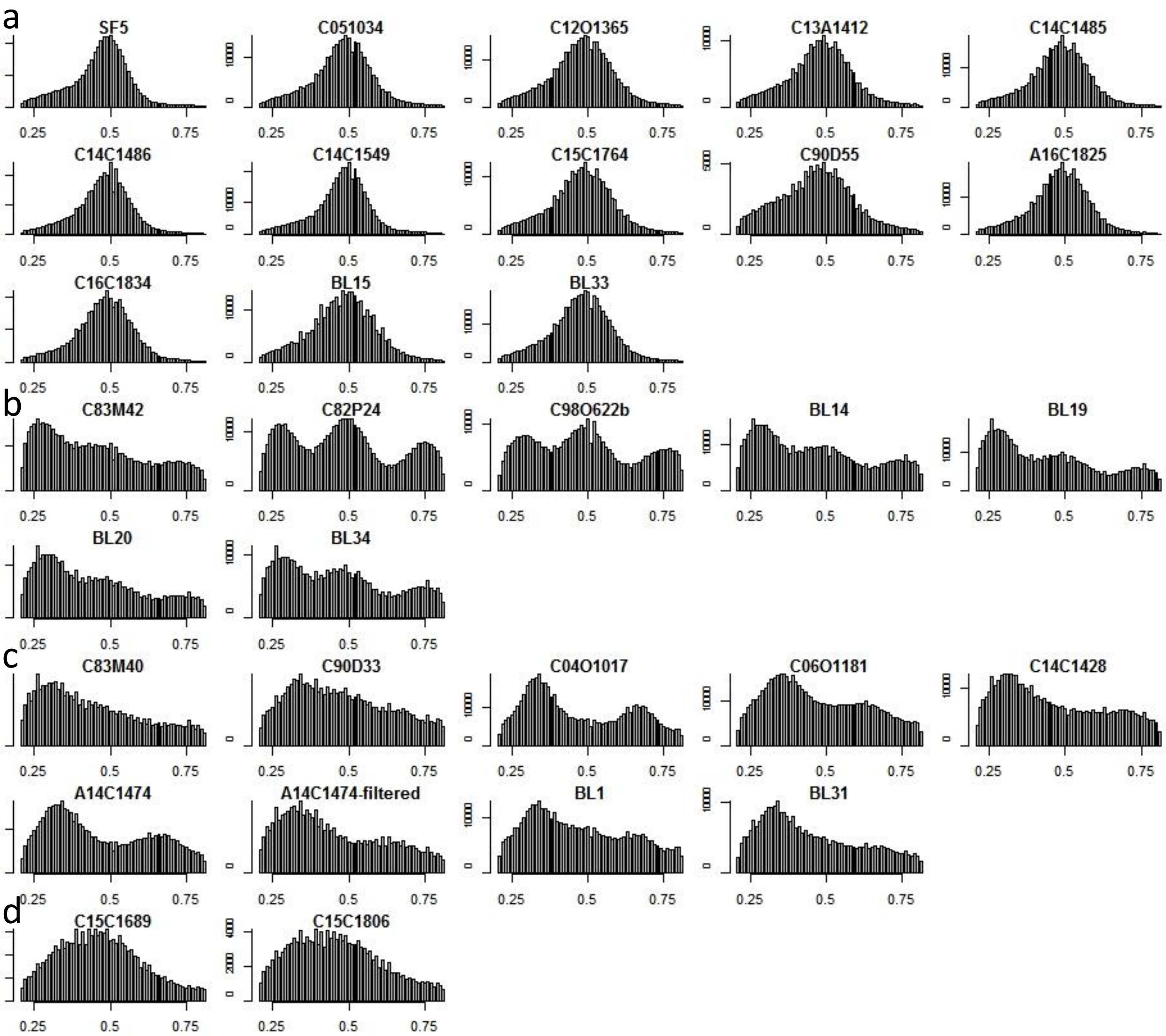

Supplementary Figure 4. Bar plots of alternative allele frequencies for each SNP detected between the tested isolate and the SF5 assembly.

The x axis defines the frequency of reads for each SNP. The y axis defines the number of SNPs with that frequency. a) Ten American isolates and three European isolates, including the reference isolate SF5 with a unimodal distribution with a peak at 0.5 indicative of a homokaryotic state. b) Three Californian and four European isolates with a trimodal distribution with peaks at 0.25, 0.5, and 0.75 consistent with a heterokaryotic with two nuclei in a 1:1 ratio. c) Six American isolates, plus one isolate which was filtered (A14C1474-filtered) and two European isolates with bimodal distributions with peaks at 0.33 and 0.67 consistent with a heterokaryon made up of three nuclei in a 1:1:1 ratio. d) Two Californian isolates had SNP frequency profiles which did not have clear unimodal, trimodal, or bimodal distributions; these could be heterokaryons containing multiple nuclei.

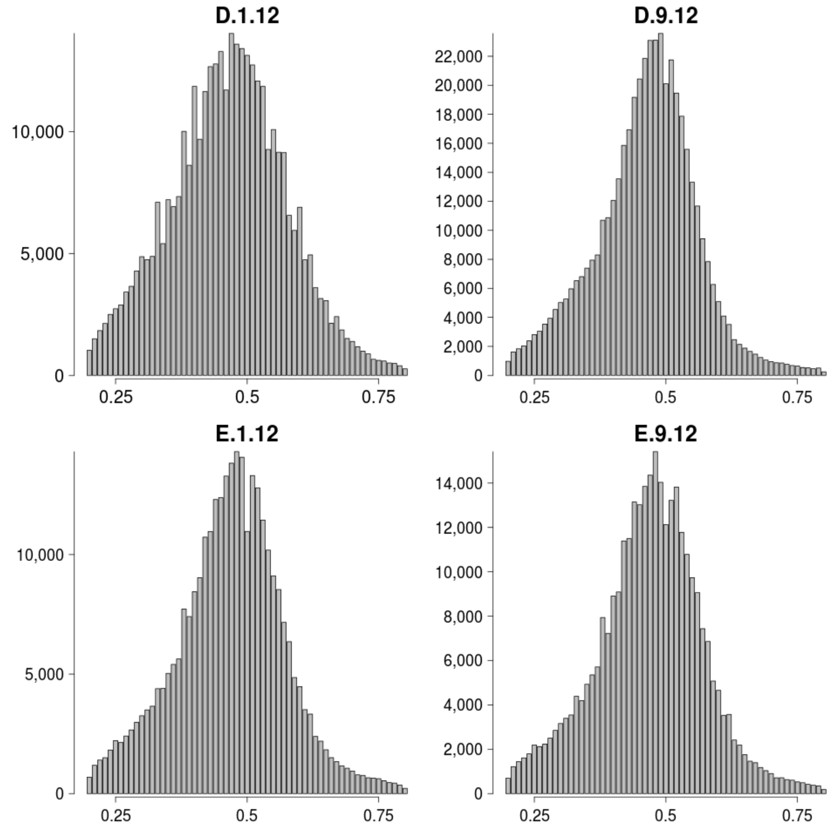

Supplementary Figure 5. Bar plots of alternative allele frequencies for each SNP detected between 4 progeny isolates and the SF5 assembly.

All 4 sexual progeny from the SF5 x C82P24 cross tested had a unimodal distribution with a peak at 0.5. This is consistent with each sexual progeny being a diploid, with a single haploid gamete being contributed from each parent.

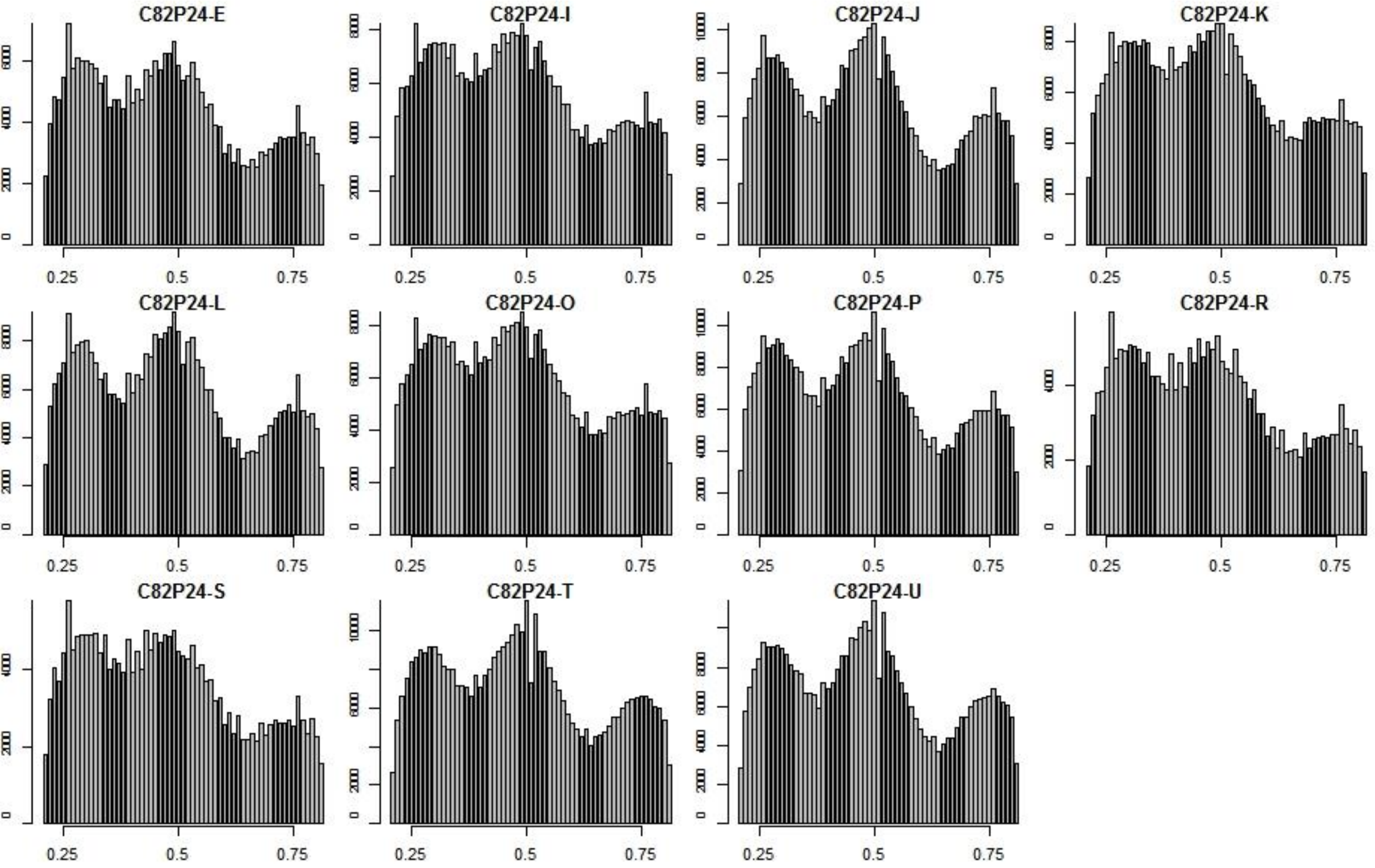

Supplementary Figure 6. Bar plots of alternative allele frequencies for each SNP detected between 11 C82P24 single spore derivatives and the SF5 assembly.

All 11 derivatives had a trimodal distribution with three peaks at 0.25, 0.5 and 0.75 consistent with each multinucleate spore transmitting two genetically distinct nuclei to the next asexual generation.

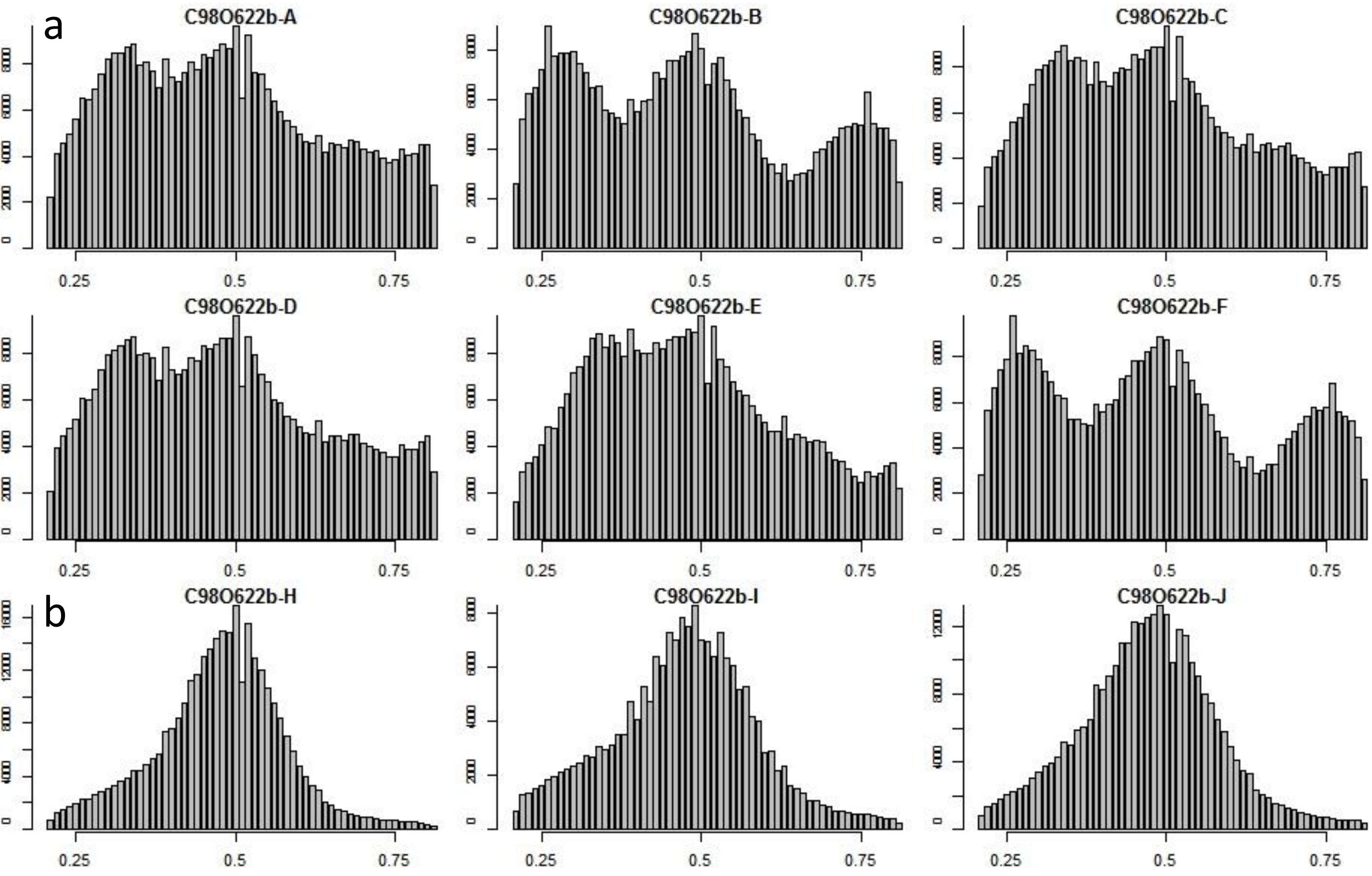

Supplementary Figure 7. Bar plots of alternative allele frequencies for each SNP detected between nine C98O622b single spore derivatives and the SF5 assembly.

a) Six of the nine derivatives had trimodal distributions; two (B and F) had peaks at 0.25, 0.5, and 0.75, while four (A, C, D & E) had peaks at 0.33, 0.5, and 0.66. b) Three of the nine derivatives had a unimodal distribution with a single peak at 0.5 consistent with the transmission of a single nuclear type through their progenitor single spores.

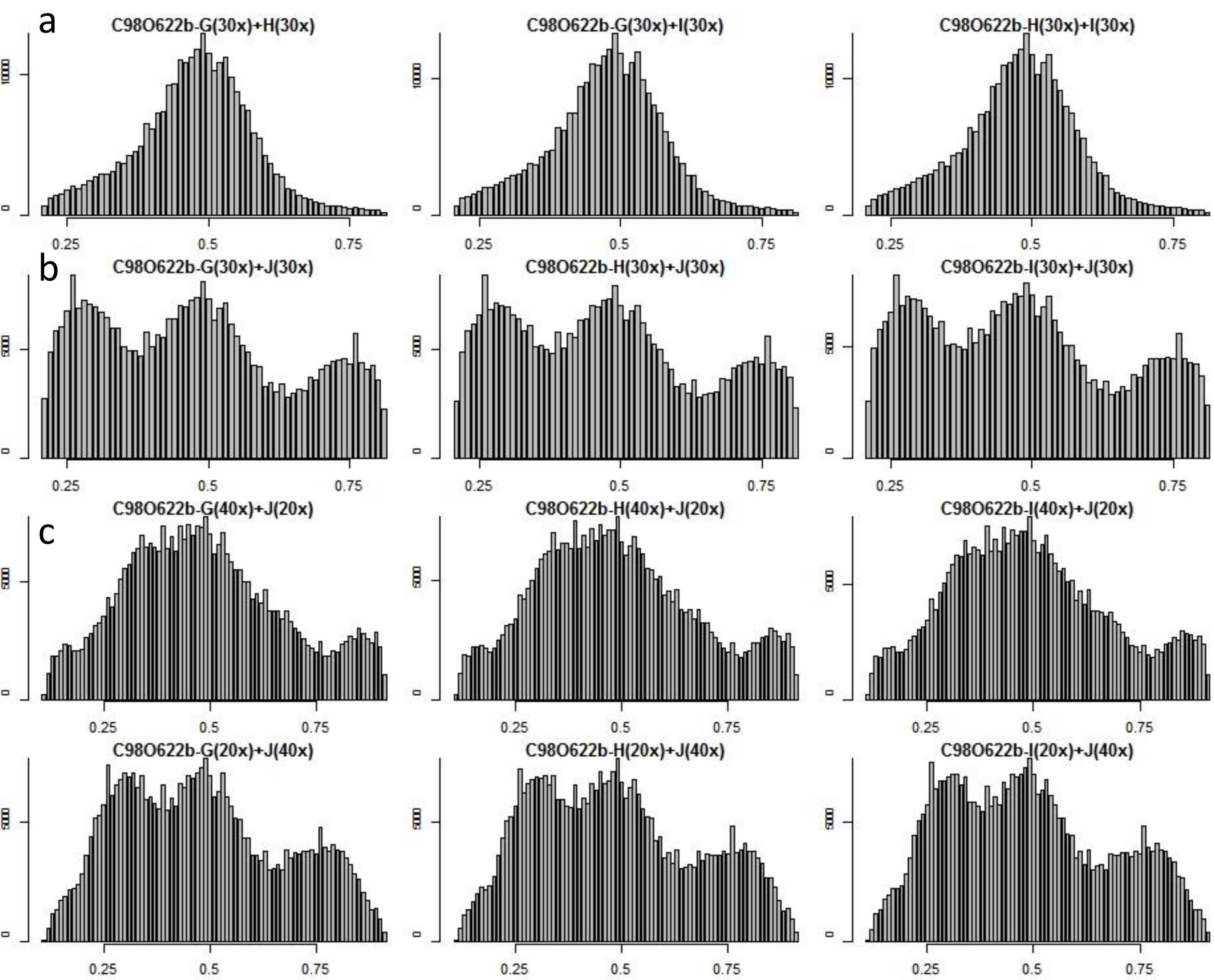

Supplementary Figure 8. Bar plots of alternative allele frequencies for each SNP detected between heterokaryons generated *in silico* and the SF5 assembly.

a) Combining reads from two homokaryons with high identity to one another produced a single peak, consistent with them being highly similar isolates, as demonstrated by kinship analysis (Figure 6). b) Combining reads from two different homokaryon nuclear types in equal combinations, simulating a 1:1 ratio produced plots with three peaks at approximately 0.25, 0.5 and 0.75, similar to C98O622b heterokaryons B and F (Figure 6, Supplementary Figure 6). c) Combining reads in 2:1 or 1:2 proportions between homokaryon nuclear types resulted in plots similar to C98O622b heterokaryons A, C, D & E (Figure 6, Supplementary Figure 6).

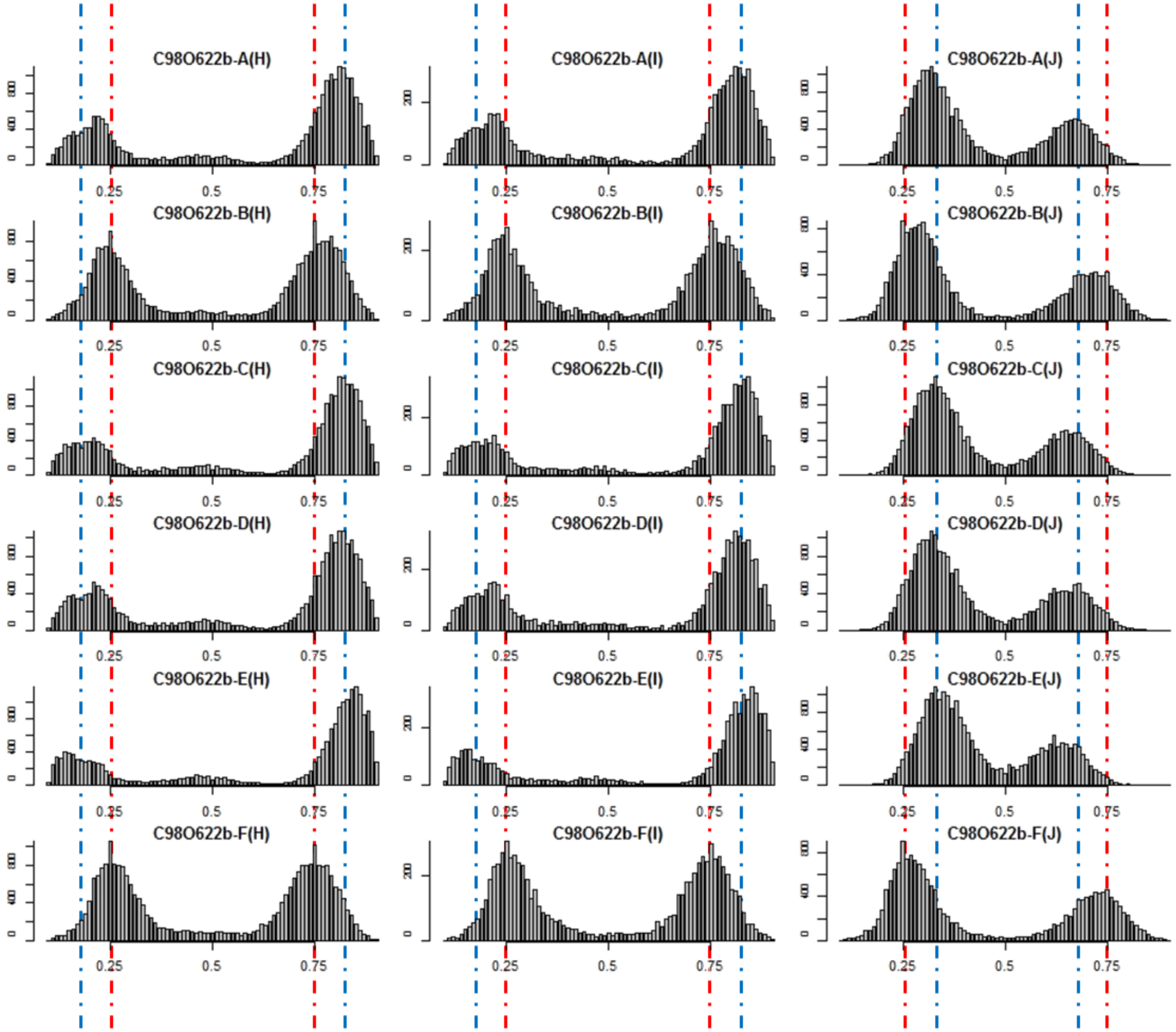

Supplementary Figure 9. Bar plots of alternative allele frequencies for SNPs unique to each homokaryotic nuclear type in the heterokaryotic derivatives and the SF5 assembly.

SNPs unique to either homokaryotic derivative nuclear type (H/I or J) were sampled from all six heterokaryotic derivatives of C98O622b. In heterokaryotic derivatives B and F (second and sixth row) with peaks previously at 0.25, 0.5 and 0.75 (Figure 6c, Supplementary Figure 7), all SNPs were detected at approximately 0.25 and 0.75 (indicated by red dotted lines). This is consistent with genetically distinct nuclei being present in a 1:1 ratio. In heterokaryons A, C, D & E, where peaks were not at 0.25, 0.5 and 0.75 (Figure 6c, Supplementary Figure 7), SNPs unique to derivative H or I (column two) were consistently at 0.17 and 0.83 (blue dotted line) while SNPs unique to J (column three) were at 0.33 and 0.67 (blue dotted line). This pattern is consistent with the second nucleus being present twice as often as the first nucleus in these heterokaryotic derivatives.

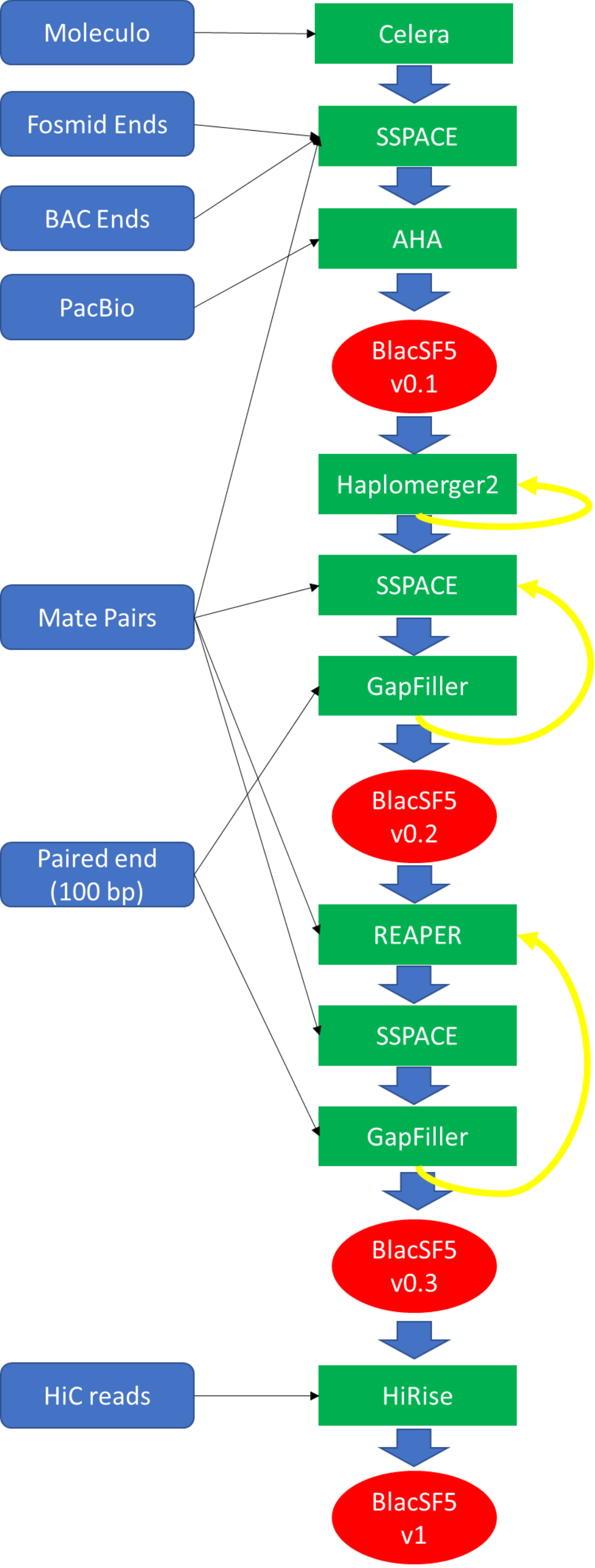

Supplementary Figure 10a. Assembly workflow used to achieve the final *B. lactucae* SF5 assembly.

Blue boxes are input data. Green boxes are software used to incorporate the data. Red circles are checkpoints assessed in Supplementary Figure 10b. Black arrows connect input data to the software used. Blue arrows show the linear progression through the workflow. Yellow arrows indicate where software(s) was applied iteratively until minimal changes were detected between input and output assembly statistics.

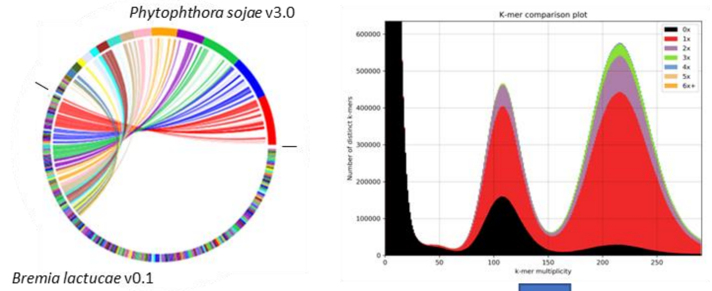

3073 scaffolds
8841 contigs
142 Kb SN_{50}
16 Kb CN_{50}
141 Mb
23% gaps
99.1% BUSCO complete
20.5% BUSCO duplicated
0% BUSCO missing

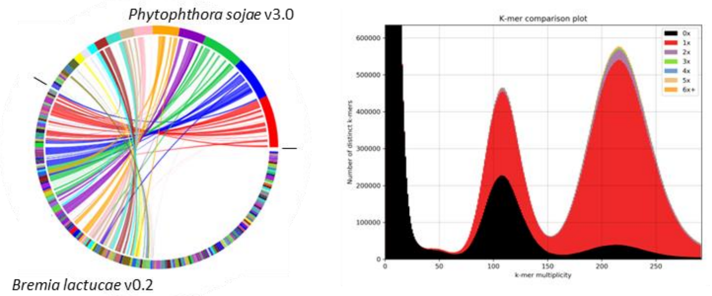

667 scaffolds
4644 contigs
529 Kb SN_{50}
33 Kb CN_{50}
117 Mb
22% gaps
97.8% BUSCO complete
3.4% BUSCO duplicated
0.9% BUSCO missing

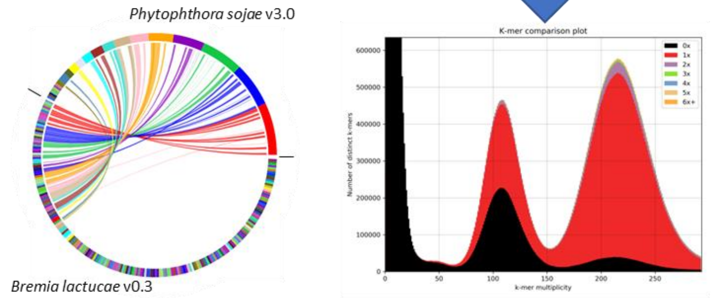

885 scaffolds
4727 contigs
284 Kb SN_{50}
31 Kb CN_{50}
116 Mb
21% gaps
98.3% BUSCO complete
3.4% BUSCO duplicated
0.8% BUSCO missing

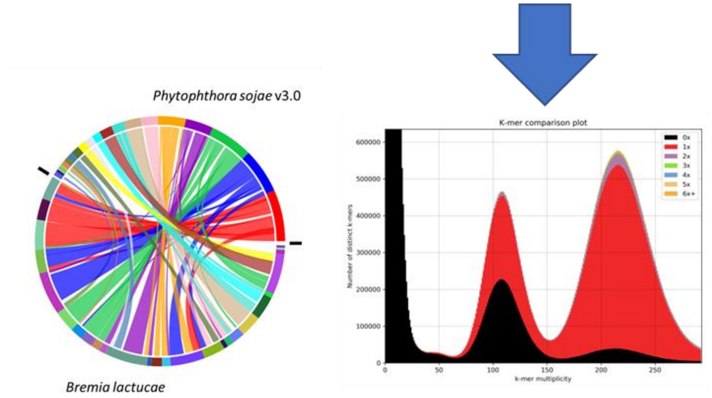

122 scaffolds
4997 contigs
6.1 Mb SN_{50}
30 Kb CN_{50}
116 Mb
21% gaps
97.8% BUSCO complete
3.4% BUSCO duplicated
0.9% BUSCO missing

Supplementary Figure 10b. Genome assembly checkpoints.

SyMap plots, displaying nuclear collinearity with *P. sojae* v3.0 became more dense as the scaffold N_{50} increased. HiRise significantly improved collinearity. KAT spectra-cn plots were used to infer redundancy within the assembly. The initial assembly had high levels of duplication, which were removed by Haplomerger2. Assembly statistics at each checkpoint are shown to the right.

Supplementary Table 1. Total nuclear DNA content of 39 *Bremia lactucae* isolates, including SF5. Sexual progeny isolates generated in the lab are italicized. Table is sorted by mean nuclear content (Mb).

Isolate label	Mean nuclear content	Standard Deviation	Replicates
A.2.12	*312.5*	*7.8*	*2*
A14C1474	*311.5*		*1*
CrAIII-19	*311.5*	*5.0*	*2*
C15C1816	310.5		1
Se6-2	*310.1*	*0.9*	*2*
C08O1181	309.6	3.7	2
SF5	307.9	4.2	16
A14C1435	307.8	3.8	4
CrAIII-18	*307.8*	*4.8*	*2*
A.5.12	*307.4*	*0.7*	*2*
V1	*306.8*	*6.6*	*2*
C04O1017	306.6	2.9	2
C05R1034	306.0	1.6	3
B.9.12	*305.8*	*2.5*	*2*
A16C1821	305.6		1
C82P24	305.4	2.4	18
ZL07A	*305.3*		*1*
C15C1806	305.2		1
C.7.12	*305.0*		*1*
CrAII-2	*304.8*		*1*
C98O648	304.5	16.0	3
C98O622	304.4	7.0	3
A.17.12	*304.2*	*2.7*	*2*
CrA III-2	*303.6*	*2.0*	*2*
C15C1771	303.3	3.8	17
C13C1414	302.8	8.5	3
C91D39	301.8	1.9	2
CrAIII-15	*301.6*	*6.9*	*2*
C83M47	301.3	1.7	2
C14C1549	301.0	5.2	2
C14C1485	300.7	3.7	4
C16C1832	300.6		1
C14C1452	298.9	9.1	3
C15C1769	298.9	4.2	6
C98O622b	296.9	8.6	3
C13A1407	294.9	7.4	3
C14C1486	294.5	3.4	6
C15C1622	291.3	12.6	4
R7	*288.8*	*8.1*	*5*
**Mean**	303.8	5.4	

Supplementary Table 2. Comparative statistics of downy mildew genome assemblies and select *Phytophthora* assemblies.

Genus	Species	Isolate/label	Scaffold N50 (kb)	Scaffold Count	Contig N50 (kb)	Contig Count	Assembly size (Mb)	Gaps (%)	Gene model count [a]	BUSCO Complete (%)	BUSCO Duplicated (%)	BUSCO Fragmented (%)	BUSCO Missing (%)	Reference
Bremia	*lactucae*	SF5	6116	122	30	4997	115.9	21.482	9781	98.3	3.4	0.9	0.8	This study
Hyaloperonospora	*arabidopsidis*	Cala2	24	9283	22	9658	70.3	0.016	n/a	96.6	1.3	3	0.4	n/a
		Emoy2	332	3044	43	10401	78.9	10.224	14321	96.6	4.7	2.6	0.8	1
		Noks1	19	12086	18	13094	74.2	0.025	n/a	97	1.3	3	0	n/a
Peronospora	*effusa*	R13	72	784	48	1472	32.2	0.261	8607	97.8	0.4	0	2.2	2
		R14	61	880	52	1275	30.8	0.564	8571	97	0	0.4	2.6	
	tabacina	968-J2	79	4016	11	10799	63.1	27.351	11310	94.9	29.5	3	2.1	3
		968-S26	61	3245	15	8552	55.3	19.089	10707	94.9	29.1	3.4	1.7	
Plasmopara	*halstedii*	Ph8-99-BlA4	1546	3162	16	25359	75.3	11.322	15469	97.4	0	1.7	0.9	4
	viticola	INRA-PV221	181	1883	49	3995	74.7	2.83	n/a	95.7	4.7	1.7	2.6	5
		JL-7-2	172	2165	14	23193	101.2	16.712	n/a (17014)	84.6	8.1	8.5	6.9	6
Pseudoperonospora	*cubensis*	ASM25260v1	4	35539	4	35539	64.3	0	n/a	92.8	0.9	6.4	0.8	7
Sclerospora	*graminicola*	UoM-SG-P1	18	26786	16	28799	299.9	0.29	n/a (38120)	86.4	12	4.3	9.3	8
		Iwate	24	64505	8	82033	253.7	6.3	n/a	92.8	0.9	6.4	0.8	9
Phytophthora	*infestans*	T30-4	1589	4921	44	18288	228.5	16.806	17797	97	3	1.3	1.7	10
	ramorum	ASM14973v1	308	2576	48	7589	66.7	18.346	15605	97.4	3	1.7	0.9	11
	sojae	Physo3	7609	83	386	863	82.6	3.959	26489	99.5	3.8	0	0.5	11

[a] n/a indicates that annotations could not be found or weren't described. Bracketed numbers indicate reported numbers from paper.

Supplementary Table 3. Annotation statistics of 25 oomycete genome assemblies in the public domain, plus calculated mean and standard deviation.

	Biotroph?	Total sequence length	Number of genes	Number of single exon genes	% single exonic	Number of exons	Number of introns	Total gene length	% of genome genic	Total exon length	% of genome exonic	Total intron length	% of genome intronic	Shortest gene	Shortest exon	Shortest intron	Longest gene	Longest exon	Longest intron	mean gene length	mean exon length	mean intron length	BUSCO % Complete	BUSCO % Duplicated	BUSCO % Fragmented	BUSCO % Missing
Albugo candida	Y	34563972	15824	10776	68.10%	23793	7969	15389934	44.53%	14741853	42.65%	664019	1.92%	150	3	21	32320	29687	1184	973	620	83	92.4	0.9	5.6	2.0
Albugo laibachii	Y	32766811	14629	5668	38.74%	44766	30141	25380733	77.46%	19508810	59.54%	5931553	18.10%	8	1	4	43352	15117	29413	1735	436	197	91.1	7.3	5.1	3.8
Aphanomyces astaci	N	75844385	19584	16926	86.43%	129725	103001	36007310	47.48%	42642424	56.22%	14329529	18.89%	64	3	11	39426	27075	14958	1839	329	139	97.0	0.4	2.6	0.4
Aphanomyces invadans	N	71402472	15416	13038	84.57%	109371	88387	28444127	39.84%	37747039	52.87%	6081288	8.52%	65	3	21	38775	26036	13621	1845	345	69	97.0	0.4	2.6	0.4
Bremia lactucae	Y	115803443	9781	5009	51.21%	22819	13038	16495418	14.24%	15169054	13.10%	1352440	1.17%	180	4	10	20723	18324	9306	1686	665	104	98.3	3.4	0.9	0.8
Hyaloperonospora arabidopsidis	Y	78897814	14267	7470	52.36%	27894	13645	15544699	19.70%	13658556	17.31%	1910916	2.42%	21	3	4	14439	14439	1797	1090	490	140	96.6	4.7	2.6	0.8
Peronospora effusa (R13)	Y	32162939	8607	4076	47.36%	20294	11687	13546486	42.12%	12152818	37.79%	1417042	4.41%	180	4	10	20029	15891	9789	1574	599	121	97.8	0.4	0.0	2.2
Peronospora effusa (R14)	Y	30813553	8571	4064	47.42%	20233	11662	13528372	43.90%	12183290	39.54%	1368406	4.44%	180	4	10	18399	18399	8418	1578	602	117	97.0	0.0	0.4	2.6
Peronospora tabacina	Y	55326288	10707	3798	35.47%	27083	16376	15748764	28.47%	13913667	25.15%	1867849	3.38%	165	3	4	22458	14844	11149	1471	514	114	94.9	29.5	3.0	2.1
Phytophthora capsici	N	64023748	19805	9944	50.21%	43610	23805	24818348	38.76%	20364086	31.81%	4501872	7.03%	150	1	1	97840	13570	48310	1253	467	189	96.6	0.0	2.6	0.8
Phytophthora cinnamomi	N	77967402	26131	13429	51.39%	54779	28648	34043544	43.66%	29347224	37.64%	4753616	6.10%	150	1	1	39410	22973	26680	1303	536	166	97.8	2.1	1.7	0.5
Phytophthora infestans	N	228543505	19344	7243	37.44%	51128	31784	28249995	12.36%	24329191	10.65%	3984372	1.74%	37	2	17	31653	31653	3672	1460	476	125	97.0	3.0	1.3	1.7
Phytophthora parasitica	N	82389172	23240	18832	81.03%	74486	46425	35403323	42.97%	44293131	53.76%	5043693	6.12%	49	2	21	36241	31698	11821	1523	595	109	97.9	0.9	1.3	0.8
Phytophthora ramorum	N	66652401	15743	7298	46.36%	40639	24896	25791090	38.69%	22490786	33.74%	3350096	5.03%	103	1	11	32670	29384	28977	1638	553	135	97.4	3.0	1.7	0.9
Phytophthora sojae	N	82597641	28142	13742	48.83%	64767	36625	37664754	45.60%	31460413	38.09%	6277591	7.60%	67	1	10	59331	18498	44179	1338	486	171	99.5	3.8	0.0	0.5
Plasmopara halstedii	Y	75321785	15469	5570	36.01%	40302	24833	20008460	26.56%	17682164	23.48%	2375962	3.15%	150	3	22	42173	14651	5181	1293	439	96	97.4	0.0	1.7	0.9
Pythium aphanidermatum	N	35876849	12363	5313	42.98%	28781	16421	19938282	55.57%	18124275	50.52%	1845134	5.14%	68	1	4	31507	13869	2836	1613	630	112	94.9	0.0	3.4	1.7
Pythium arrhenomanes	N	44672625	13857	4778	34.48%	36628	22774	21277175	47.63%	18535576	41.49%	2786476	6.24%	67	1	4	32781	20346	3105	1535	506	122	87.2	1.3	9.4	3.4
Pythium irregulare	N	42968084	13869	4869	35.11%	39801	25948	23576531	54.87%	20671147	48.11%	2953919	6.87%	44	1	4	26640	26640	3132	1700	519	114	97.5	1.3	2.6	0.0
Pythium iwayami	N	43200612	14874	5797	38.97%	41050	26175	22937494	53.10%	19758311	45.74%	3232284	7.48%	90	1	4	33176	22314	3183	1542	481	123	85.0	0.4	7.7	7.3
Pythium ultimum BR650	N	37631444	14086	2653	18.83%	51739	37643	21055984	55.95%	16123738	42.85%	5010312	13.31%	84	1	4	27808	15159	4143	1495	312	133	62.8	0.0	23.9	13.3
Pythium ultimum DAOMBR144	N	44913582	15430	6131	39.73%	40259	24797	23006129	51.22%	20165552	44.90%	2900388	6.46%	68	1	4	29510	21357	9888	1491	501	117	99.1	0.4	0.4	0.5
Pythium vexans	N	33844883	11991	4198	35.01%	35050	23066	21133503	62.44%	18855723	55.71%	2323224	6.86%	72	1	4	28727	23354	2351	1762	538	101	96.6	0.4	3.0	0.4
Saprolegnia diclina	N	62885792	17448	8411	48.21%	78951	60633	30272668	48.14%	28722991	45.67%	3697283	5.88%	65	3	20	39156	26039	7242	1735	364	61	97.1	0.9	1.7	1.2
Saprolegnia parasitica	N	53131624	20435	5986	29.29%	80699	60264	30698349	57.78%	27191005	51.18%	3627872	6.83%	62	1	4	30543	16034	5440	1502	337	60	93.6	6.0	4.7	1.7
Average		64168113	15985	7801	48.80%	49146	32426	23998459	37.40%	22393313	34.90%	3743485	5.83%	94	2	9	34763	21094	12391	1519	494	121				
StDev		39673010	4852	4236		26891	22938	7035914		8775547		2660755		52	1	7	15839	5905	12768	214	97	34				
Average biotroph		56957076	12232	5804	47.45%	28398	16169	16955358	29.77%	14876277	26.12%	2111023	3.71%	129	3	11	26737	17669	9530	1425	546	122				
StDev biotroph		28872708	2919	2186		8633	7019	3704405		2411328		1519382		67	1	7	10403	4777	8274	261	82	33				
Average non-biotroph		67561542	17750	8740	49.24%	58910	40076	27312859	40.43%	25930742	38.38%	4511703	6.68%	77	1	9	38541	22706	13738	1563	469	120				
StDev non-biotroph		43432078	4568	4623		27026	23826	5658050		8450427		2735068		31	1	7	16535	5693	14208	170	94	35				

Supplementary Table 4. Orthology of previously reported candidate effectors of *B. lactucae* to proteins annotated in the SF5 genome.

Orthogroup	Stassen *et al.* 2013 / Giesbers *et al*. 2017 / Pelgrom *et al*. 2018	SF5 assembly (this paper)
1	BLR17	BlacSF5_002585-RA
	BLR28	BlacSF5_003650-RA
	BLR33	BlacSF5_009425-RA
2	BLR19	BlacSF5_006115-RA
	**BLR31**	BlacSF5_008983-RA
	**BLR40**	
3	BLQ01	BlacSF5_008167-RA
		BlacSF5_008168-RA
		BlacSF5_008173-RA
		BlacSF5_009528-RA
4	BLG02	BlacSF5_002342-RA
	**BLG03**	BlacSF5_004206-RA
5	BLR09	BlacSF5_007121-RA
	BLR10	BlacSF5_007131-RA
6	BLR11	BlacSF5_004761-RA
	BLR35 (3)	BlacSF5_004760-RA (3)
7	BLR20 (1)	BlacSF5_003745-RA
		BlacSF5_003751-RA
		BlacSF5_003753-RA
8	**BLN08 (4)**	BlacSF5_000769-RA (2)
	BLR36 (5)	BlacSF5_003408-RA (3)
9	BLN01 (7)	BlacSF5_008217-RA (7)
	**BLN06 (6)**	BlacSF5_008226-RA (5)
10	BLR05	BlacSF5_006000-RA
		BlacSF5_006001-RA
11	BLR15	BlacSF5_005890-RA
	BLR30	
12	BLR16	BlacSF5_005893-RA
		BlacSF5_009559-RA
13	BLR18	BlacSF5_002279-RA
		BlacSF5_002280-RA
14	BLR25	BlacSF5_004523-RA
		BlacSF5_006920-RA
15	BLR32	BlacSF5_008977-RA
		BlacSF5_008980-RA
16	**BLR38**	BlacSF5_005743-RA
		BlacSF5_005744-RA
17	BLC01	BlacSF5_001066-RA
		BlacSF5_009260-RA
18	BLN05	BlacSF5_002598-RA
	BLQ04	
19	**BLG01**	BlacSF5_006965-RA

20	BLR02	BlacSF5_002587-RA
21	BLR06	BlacSF5_002950-RA
22	BLR07 (4)	BlacSF5_008762-RA (3)
23	BLR13 (5)	BlacSF5_002525-RA (3)
24	BLR23	BlacSF5_008952-RA
25	BLR27	BlacSF5_002491-RA
26	BLR37	BlacSF5_008452-RA (5)
No orthology in SF5 assembly	BLR01	
	BLR03	
	BLR04	
	BLR08	
	BLR12	
	BLR14	
	BLR21	
	BLR22	
	BLR24	
	BLR26	
	BLR29	
	BLN03	
	BLN04	234 candidate effectors novel to SF5 assembly

Key:

Red: Contains WY (# domains detected)

Bold: Effector response detected

BLR	RxLR motif present
BLQ	QxLR motif present
BLN	RxLR not detected
BLC	Crinkler

Supplementary Table 5. Pathogenicity domains annotated in genome assemblies of Peronosporales spp.

	Bremia lactucae	*Peronospora*				*Hyaloperonospora arabidopsidis*	*Plasmopara halstedii*	*Phytophthora*			Downy mildews *vs.* *Phytophthora* spp.	*Phytophthora* spp. + *P. halstedii* + *B. lactucae* *vs.* Peronosporales lineage
		effusa		*tabacina*								
	SF5	R13	R14	J2	S26			*sojae*	*infestans*	*ramorum*	**t-test**	**t-test**
Serine protease	27	14	13	21	12	12	27	42	37	36		<0.05
Aspartic protease	11	11	9	17	7	15	26	147	20	116	<0.05	
Cysteine protease	10	16	17	18	17	16	20	28	27	30		
Metalloprotease	18	16	18	14	16	25	17	38	24	26		
Kazal-like serine protease inhibitor	3	1	1	2	2	7	17	45	35	17	<0.01	<0.01
Cystatin-like cysteine protease inhibitor	1	1	1	1	1	1	3	4	6	4	<0.01	<0.05
Cutinase	0	0	0	0	0	2	2	15	4	4	<0.01	
Pectin lyase	14	11	13	6	8	22	19	122	100	82	<0.001	<0.05
CAP domain	41	39	42	45	48	38	72	155	112	104	<0.01	
NPP1-like	13	9	10	17	14	21	19	80	28	62	<0.01	
Elicitin-like	20	16	15	12	8	20	20	77	54	61	<0.001	<0.05
Jacalin	15	10	10	7	7	10	20	31	22	27		<0.05
Frequency	0.0177	0.0168	0.0173	0.0141	0.013	0.0132	0.0167	0.0295	0.0262	0.0361	<0.001	<0.05

Supplementary Table 6. Nuclear DNA content of 12 asexual single sporangia derivatives of *B. lactucae* isolate C82P24.

Derivative	Nuclear DNA content
P24 A	304.2
P24 B	304.8
P24 C	302.0
P24 D	305.7
P24 E	308.5
P24 F	301.7
P24 G	301.3
P24 H	297.5
P24 I	301.8
P24 J	301.2
P24 K	308.9
P24 L	300.9
**Mean**	303.2 s.d. +/-3.3

Supplementary Table 7. Collection dates and geographic regions of *B. lactucae* diversity panel isolates sequenced in this study.

Isolate	Collection date	Collection location	Homokaryon?
SF5	~ 1975	Europe	Y
C82P24	1982	California	N
C83M42	1983	California	N
C83M40	1983	California	N
C90D55	1990	California	Y
C90D33	1990	California	N
C98O622b	1998	California	N
C04O1017	2004	California	N
C05O1034	2005	California	Y
C06O1181	2006	California	N
C12O1365	2012	California	Y
C13A1412	2013	California	Y
C14C1485	2014	California	Y
C14C1486	2014	California	Y
C14C1549	2014	California	Y
C14C1428	2014	California	N
A14C1474	2014	Arizona	N
A14C1474-filtered	2014	Arizona	N
C15C1764	2015	California	Y
C15C1689	2015	California	N
C15C1806	2015	California	N
A16C1825	2016	Arizona	Y
C16C1834	2016	California	Y
BL15	1980's*	Europe	Y
BL33	2017*	Europe	Y
BL14	1980's*	Europe	N
BL19	1999*	Europe	N
BL20	1999*	Europe	N
BL34	2018*	Europe	N
BL1	< 1980*	Europe	N
BL31	2013*	Europe	N

*Date reported for European isolate reflects year of nomination, not isolation.

Supplementary Table 8. Sequencing statistics for *B. lactucae* isolates, including number of reads sequenced, percent that map to the SF5 assembly, coverage and under which BioProject they may be found.

Isolate	Sequencing tech	Number of reads	% of reads mapped	Coverage	Notes	BioProject
SF5	Illumina	381857728	46.47%	164x	Paired end	PRJNA387613
SF5	Moleculo (12f8)	2900	99.55%	<1x	Synthetic long read	PRJNA387613
SF5	Moleculo (31-1366)	84441	99.57%	3x	Synthetic long read	PRJNA387613
SF5	Moleculo (32-1815)	111413	99.47%	5x	Synthetic long read	PRJNA387613
SF5	Moleculo (32-1820)	102453	99.46%	4x	Synthetic long read	PRJNA387613
SF5	PacBio	997567	78.96%	13x	Single molecule sequencing	PRJNA387613
SF5	Hi-C	921209398	74.46%	700x	Paired end Hi-C	PRJNA387613
SF5	Illumina	2662552	67.54%	2x	Mate pair	PRJNA387613
SF5	Illumina	10003434	95.69%	12x	Mate pair	PRJNA387613
SF5	Illumina	7513028	95.84%	10x	Mate pair	PRJNA387613
SF5	Illumina	6201454	95.64%	8x	Mate pair	PRJNA387613
SF5	Illumina	5053300	94.34%	6x	Mate pair	PRJNA387613
C82P24	Illumina	292885800	51.33%	133x	Paired end	PRJNA387613
C04O1017	Illumina	124562222	59.47%	84x	Paired end	PRJNA387192
C05O1034	Illumina	152094538	36.82%	63x	Paired end	PRJNA387192
C06O1181	Illumina	196926958	51.48%	119x	Paired end	PRJNA387192
C12O1365	Illumina	230017936	24.96%	61x	Paired end	PRJNA387192
C13A1412	Illumina	119679636	40.63%	55x	Paired end	PRJNA387192
C14C1428	Illumina	152305120	37.60%	62x	Paired end	PRJNA387192
A14C1474	Illumina	140096428	53.97%	86x	Paired end	PRJNA387192
A14C1474-filtered	Illumina	158928356	27.07%	48x	Paired end	PRJNA387192
C14C1485	Illumina	192403228	25.98%	57x	Paired end	PRJNA387192
C14C1486	Illumina	317858696	21.49%	78x	Paired end	PRJNA387192
C14C1549	Illumina	147766830	55.92%	97x	Paired end	PRJNA387192
C15C1689	Illumina	161185662	27.47%	50x	Paired end	PRJNA387192
C15C1806	Illumina	121971270	33.03%	44x	Paired end	PRJNA387192
C98O622b	Illumina	198372578	37.22%	86x	Paired end	PRJNA387192

BL1	Illumina	237723414	29.29%	71x	Paired end	PRJNA387192
BL14	Illumina	131951684	47.62%	68x	Paired end	PRJNA387192
BL31	Illumina	164192588	35.25%	44x	Paired end	PRJNA387192
C83M40	Illumina	153187866	29.38%	48x	Paired end	PRJNA387192
C83M42	Illumina	188644422	35.99%	77x	Paired end	PRJNA387192
C90D33	Illumina	106988332	48.52%	58x	Paired end	PRJNA387192
C90D55	Illumina	188644422	22.41%	45x	Paired end	PRJNA387192
A16C1825	Illumina	91492454	55.63%	57x	Paired end	PRJNA387192
C16C1834	Illumina	98033458	54.23%	60x	Paired end	PRJNA387192
BL15	Illumina	117016930	39.20%	42x	Paired end	PRJNA387192
BL33	Illumina	164627762	34.53%	55x	Paired end	PRJNA387192
BL19	Illumina	165643306	35.18%	63x	Paired end	PRJNA387192
BL20	Illumina	132377980	37.51%	55x	Paired end	PRJNA387192
BL34	Illumina	90760346	72.21%	64x	Paired end	PRJNA387192
C98O622b(A)	Illumina	156925864	44.64%	73x	Paired end	PRJNA387017
C98O622b(B)	Illumina	156978090	32.17%	62x	Paired end	PRJNA387017
C98O622b(C)	Illumina	155602418	44.92%	75x	Paired end	PRJNA387017
C98O622b(D)	Illumina	156089754	42.87%	71x	Paired end	PRJNA387017
C98O622b(E)	Illumina	137431390	48.53%	72x	Paired end	PRJNA387017
C98O622b(F)	Illumina	166763142	37.74%	65x	Paired end	PRJNA387017
C98O622b(G)	Illumina	145772678	27.32%	39x	Paired end	PRJNA387017
C98O622b(H)	Illumina	152878822	44.47%	72x	Paired end	PRJNA387017
C98O622b(I)	Illumina	165453418	29.06%	51x	Paired end	PRJNA387017
C98O622b(J)	Illumina	160013246	38.34%	65x	Paired end	PRJNA387017
A.1.12	Illumina	43797472	64.20%	25x	Paired end	PRJNA387454
A.14.12	Illumina	51426500	51.67%	22x	Paired end	PRJNA387454
A.16.12	Illumina	104698120	31.53%	15x	Paired end	PRJNA387454
A.19.12	Illumina	58061530	39.23%	16x	Paired end	PRJNA387454
A.6.12	Illumina	213442758	19.11%	35x	Paired end	PRJNA387454
A.9.12	Illumina	63438208	43.24%	20x	Paired end	PRJNA387454
B.1.12	Illumina	36418432	49.21%	12x	Paired end	PRJNA387454

B.2.12	Illumina	66165762	49.55%	25x	Paired end	PRJNA387454
BS	Illumina	59109738	15.13%	9x	Paired end	PRJNA387454
C.11.12	Illumina	133290666	27.10%	40x	Paired end	PRJNA387454
C.15.12	Illumina	48808646	25.46%	13x	Paired end	PRJNA387454
C.17.12	Illumina	136790610	27.46%	27x	Paired end	PRJNA387454
C.18.12	Illumina	271602394	8.76%	19x	Paired end	PRJNA387454
C20	Illumina	121223828	33.37%	39x	Paired end	PRJNA387454
C.6.12	Illumina	19737042	52.53%	10x	Paired end	PRJNA387454
C.8.12	Illumina	55698218	25.84%	16x	Paired end	PRJNA387454
C.9.12	Illumina	82118898	31.60%	29x	Paired end	PRJNA387454
D10	Illumina	31528018	52.61%	13x	Paired end	PRJNA387454
D.1.12	Illumina	234551168	19.22%	49x	Paired end	PRJNA387454
D.12.12	Illumina	66740438	29.05%	21x	Paired end	PRJNA387454
D.13.12	Illumina	38717330	40.63%	19x	Paired end	PRJNA387454
D.17.12	Illumina	34188028	57.00%	23x	Paired end	PRJNA387454
D.2.12	Illumina	53547244	43.94%	20x	Paired end	PRJNA387454
D.3.12	Illumina	21753776	63.05%	10x	Paired end	PRJNA387454
D.5.12	Illumina	31055756	48.66%	17x	Paired end	PRJNA387454
D.6.12	Illumina	65280916	24.20%	12x	Paired end	PRJNA387454
D.8.12	Illumina	107964364	18.29%	14x	Paired end	PRJNA387454
D.9.12	Illumina	232549460	38.81%	72x	Paired end	PRJNA387454
E11	Illumina	68504354	47.63%	24x	Paired end	PRJNA387454
E.11.12	Illumina	53521506	29.00%	17x	Paired end	PRJNA387454
E.1.12	Illumina	173974472	30.79%	54x	Paired end	PRJNA387454
E.5.12	Illumina	155218938	15.45%	16x	Paired end	PRJNA387454
E.9.12	Illumina	198725368	23.88%	57x	Paired end	PRJNA387454
J10	Illumina	58518472	55.49%	29x	Paired end	PRJNA387454
R10	Illumina	155923800	30.31%	36x	Paired end	PRJNA387454
S11	Illumina	112705706	18.40%	15x	Paired end	PRJNA387454
Y1	Illumina	82883272	58.10%	37x	Paired end	PRJNA387454
Y10	Illumina	6995742	67.48%	7x	Paired end	PRJNA387454

C82P24(B)	Illumina	37025082	67.63%	27x	Paired end	PRJNA387017
C82P24(C)	Illumina	68928676	49.91%	37x	Paired end	PRJNA387017
C82P24(D)	Illumina	52385436	70.13%	40x	Paired end	PRJNA387017
C82P24(E)	Illumina	75232112	63.88%	52x	Paired end	PRJNA387017
C82P24(F)	Illumina	43699908	43.42%	20x	Paired end	PRJNA387017
C82P24(G)	Illumina	58391208	75.25%	48x	Paired end	PRJNA387017
C82P24(H)	Illumina	74366634	55.24%	45x	Paired end	PRJNA387017
C82P24(I)	Illumina	79943608	68.07%	60x	Paired end	PRJNA387017
C82P24(J)	Illumina	79573208	75.63%	67x	Paired end	PRJNA387017
C82P24(K)	Illumina	106798776	55.34%	65x	Paired end	PRJNA387017
C82P24(L)	Illumina	106901626	51.70%	61x	Paired end	PRJNA387017
C82P24(M)	Illumina	68527398	58.36%	43x	Paired end	PRJNA387017
C82P24(N)	Illumina	74032176	56.83%	45x	Paired end	PRJNA387017
C82P24(O)	Illumina	106872988	52.19%	61x	Paired end	PRJNA387017
C82P24(P)	Illumina	83314294	77.66%	71x	Paired end	PRJNA387017
C82P24(Q)	Illumina	65370276	53.28%	38x	Paired end	PRJNA387017
C82P24(R)	Illumina	70495622	64.93%	50x	Paired end	PRJNA387017
C82P24(S)	Illumina	80405436	56.38%	49x	Paired end	PRJNA387017
C82P24(T)	Illumina	110186616	66.32%	81x	Paired end	PRJNA387017
C82P24(U)	Illumina	105981740	64.18%	75x	Paired end	PRJNA387017
SF5(A)	Illumina	84003116	63.70%	56x	Paired end	PRJNA387017
SF5(B)	Illumina	93533616	71.66%	69x	Paired end	PRJNA387017
SF5(C)	Illumina	109630452	50.88%	57x	Paired end	PRJNA387017

Supplementary Table 9. SRA accession numbers for oomycete sequences downloaded for this study.

Genus	Species	Strain	SRA #
Albugo	*candida*	Nc2	SRR1811450, SRR1811464
Albugo	*candida*	BoL	SRR1811474
Albugo	*candida*	BoT	SRR1811472, SRR1811473
Albugo	*candida*	2v	SRR1811471
Albugo	*candida*	EM2	SRR1806791
Albugo	*laibachii*	Nc14	ERR022981, ERR022982
Aphanomyces	*astaci*	APO3	SRR957817
Aphanomyces	*invadans*	NJM9701	SRR957818
Hyaloperonospora	*arabidopsidis*	Emoy2	ERR012782-ERR012787, ERR015788, ERR015788
Hyaloperonospora	*arabidopsidis*	Cala2	SRR3254743
Hyaloperonospora	*arabidopsidis*	Noks1	SRR3254744
Peronospora	*effusa*	R14	SRR7107520
Phytophthora	*agathidicida*	NZFS3770	SRR2124796
Phytophthora	*agathidicida*	NZF3772	SRR2124795
Phytophthora	*capsici*	Pc33E	SRR5436369
Phytophthora	*capsici*	PC1E8	SRR5436366
Phytophthora	*infestans*	P3683	SRR1817202
Phytophthora	*infestans*	PIC99189	ERR1990236
Phytophthora	*nicotianae*	R0	SRR2245478
Phytophthora	*nicotianae*	R1	SRR2198696
Phytophthora	*parasitica*	P1569	SRR653623
Phytophthora	*parasitica*	P. Nic Tob race 0	SRR653713
Phytophthora	*parasitica*	P10297	SRR714753
Phytophthora	*parasitica*	P1976	SRR653714
Phytophthora	*ramorum*	Pr-106	SRR6015292
Phytophthora	*ramorum*	WSU115-0118	SRR6015284
Phytophthora	*ramorum*	WSU108-0021	SRR6015282
Phytophthora	*ramorum*	TKTK001E	SRR2141229
Phytophthora	*ramorum*	TKTK001C	SRR2141227
Phytophthora	*ramorum*	CC1048	SRR4309669
Phytophthora	*sojae*	P7076	SRR1046800
Phytophthora	*sojae*	ACR10	SRR1046799
Pythium	*arrhenomanes*	PAR_AB	SRR234858
Pythium	*irregulare*	CBS 250.28	SRR234877
Pythium	*iwayami*	P174	SRR235482
Pythium	*ultimum var. sporangiiferum*	CBS219.65	SRR235484
Saprolegnia	*diclina*	VS20	SRR576639
Saprolegnia	*parasitica*	N12	SRR515950
Saprolegnia	*parasitica*	CBS223.65	SRR058717
Sclerospora	*graminicola*	Sg-4	DRR097254
Sclerospora	*graminicola*	UoM-SG-Pathotype1	SRR3658180

Supplementary References

1. Baxter, L. *et al.* Signatures of adaptation to obligate biotrophy in the Hyaloperonospora arabidopsidis genome. *Science* **330**(2010).
2. Fletcher, K. *et al.* Comparative genomics of downy mildews reveals potential adaptations to biotrophy. *BMC Genomics* **19**(2018).
3. Derevnina, L. *et al.* Genome Sequence and Architecture of the Tobacco Downy Mildew Pathogen Peronospora tabacina. *Molecular Plant-Microbe Interactions* **28**, 1198-1215 (2015).
4. Sharma, R. *et al.* Genome analyses of the sunflower pathogen Plasmopara halstedii provide insights into effector evolution in downy mildews and Phytophthora. *BMC Genomics* **16**, 741 (2015).
5. Dussert, Y. *et al.* Draft genome sequence of Plasmopara viticola, the grapevine downy mildew pathogen. *Genome announcements* **4**, e00987-16 (2016).
6. Yin, L. *et al.* Genome sequence of Plasmopara viticola and insight into the pathogenic mechanism. *Scientific Reports* **7**(2017).
7. Tian, M. *et al.* 454 Genome Sequencing of Pseudoperonospora cubensis Reveals Effector Proteins with a QXLR Translocation Motif. *Molecular Plant-Microbe Interactions* **24**, 543-553 (2011).
8. Kobayashi, M. *et al.* Genome analysis of the foxtail millet pathogen Sclerospora graminicola reveals the complex effector repertoire of graminicolous downy mildews. *BMC Genomics* **18**, 897 (2017).
9. Nayaka, S.C. *et al.* Draft genome sequence of Sclerospora graminicola, the pearl millet downy mildew pathogen. *Biotechnology Reports* (2017).
10. Haas, B.J. *et al.* Genome sequence and analysis of the Irish potato famine pathogen Phytophthora infestans. *Nature* **461**, 393-398 (2009).
11. Tyler, B.M. *et al.* Phytophthora genome sequences uncover evolutionary origins and mechanisms of pathogenesis. *Science* **313**(2006).
