# Supplementary material for: Genomic signatures of heterokaryosis in the oomycete pathogen Bremia lactucae
Source: Nat Commun. 2019 Jun 14;10:2645. doi: 10.1038/s41467-019-10550-0 (PMC6570648; doi:10.1038/s41467-019-10550-0)
Supplement: Supplementary file 3 — Reporting Summary [file 41467_2019_10550_MOESM3_ESM.pdf]

nature research

Corresponding author(s): Michelmore

Reporting Summary

Nature Research wishes to improve the reproducibility of the work that we publish. This form provides structure for consistency and transparency in reporting. For further information on Nature Research policies, see Authors & Referees and the Editorial Policy Checklist.

Statistical parameters

When statistical analyses are reported, confirm that the following items are present in the relevant location (e.g. figure legend, table legend, main text, or Methods section).

n/a	Confirmed	
☐	☒	The exact sample size (*n*) for each experimental group/condition, given as a discrete number and unit of measurement
☐	☒	An indication of whether measurements were taken from distinct samples or whether the same sample was measured repeatedly
☐	☒	The statistical test(s) used AND whether they are one- or two-sided<br>*Only common tests should be described solely by name; describe more complex techniques in the Methods section.*
☒	☐	A description of all covariates tested
☒	☐	A description of any assumptions or corrections, such as tests of normality and adjustment for multiple comparisons
☐	☒	A full description of the statistics including central tendency (e.g. means) or other basic estimates (e.g. regression coefficient) AND variation (e.g. standard deviation) or associated estimates of uncertainty (e.g. confidence intervals)
☐	☒	For null hypothesis testing, the test statistic (e.g. *F*, *t*, *r*) with confidence intervals, effect sizes, degrees of freedom and *P* value noted<br>*Give P values as exact values whenever suitable.*
☒	☐	For Bayesian analysis, information on the choice of priors and Markov chain Monte Carlo settings
☒	☐	For hierarchical and complex designs, identification of the appropriate level for tests and full reporting of outcomes
☒	☐	Estimates of effect sizes (e.g. Cohen's *d*, Pearson's *r*), indicating how they were calculated
☐	☒	Clearly defined error bars<br>*State explicitly what error bars represent (e.g. SD, SE, CI)*

Our web collection on statistics for biologists may be useful.

Software and code

Policy information about availability of computer code

Data collection	All software is defined and cited in the text.
Data analysis	All software is defined and cited in the text.

For manuscripts utilizing custom algorithms or software that are central to the research but not yet described in published literature, software must be made available to editors/reviewers upon request. We strongly encourage code deposition in a community repository (e.g. GitHub). See the Nature Research guidelines for submitting code & software for further information.

Data

Policy information about availability of data

All manuscripts must include a data availability statement. This statement should provide the following information, where applicable:

- Accession codes, unique identifiers, or web links for publicly available datasets
- A list of figures that have associated raw data
- A description of any restrictions on data availability

Data availability: All sequence data are available at NCBI under the following BioProjects: PRJNA387017 Bremia lactucae asexual single spore progeny WGS
PRJNA387192 Bremia lactucae diversity panel WGS
PRJNA387454 Bremia lactucae SF5 x C82P24 progeny WGS

PRJNA387613 Bremia lactucae whole genome sequencing and de novo assembly
PRJNA523226 RNAseq of lettuce infected with Bremia lactucae.
Source data underlying figures 1c, 2, 5, 6a and 7 are provided as a Source Data file.

Field-specific reporting

Please select the best fit for your research. If you are not sure, read the appropriate sections before making your selection.

☒ Life sciences ☐ Behavioural & social sciences ☐ Ecological, evolutionary & environmental sciences

For a reference copy of the document with all sections, see nature.com/authors/policies/ReportingSummary-flat.pdf

Life sciences study design

All studies must disclose on these points even when the disclosure is negative.

Sample size	n/a
Data exclusions	n/a
Replication	n/a
Randomization	n/a
Blinding	n/a

Reporting for specific materials, systems and methods

Materials & experimental systems

n/a	Involved in the study
☒	☐ Unique biological materials
☒	☐ Antibodies
☒	☐ Eukaryotic cell lines
☒	☐ Palaeontology
☒	☐ Animals and other organisms
☒	☐ Human research participants

Methods

n/a	Involved in the study
☒	☐ ChIP-seq
☐	☒ Flow cytometry
☒	☐ MRI-based neuroimaging

Flow Cytometry

Plots

Confirm that:

☒ The axis labels state the marker and fluorochrome used (e.g. CD4-FITC).

☒ The axis scales are clearly visible. Include numbers along axes only for bottom left plot of group (a 'group' is an analysis of identical markers).

☒ All plots are contour plots with outliers or pseudocolor plots.

☒ A numerical value for number of cells or percentage (with statistics) is provided.

Methodology

Sample preparation	Flow cytometry of select Bremia lactucae isolates was performed on sporulating cotyledons 7 days post iinfection. For each measurement, two sporulating cotyledons were mixed with 1 cm2 of young leaf tissue from Oryza sativa cv. Kitaake and cut in small pieces using a razor blades. Nuclei were then filtered through a 10um mesh. Nuclei were stained with propidium iodide following the CyStain PI absolute P kit (Sysmex, USA).
Instrument	BD FACScan (Becton Dickinson, East Rutherford, NJ)
Software	FlowJo (Ashland, OR)

Cell population abundance	10,000 nuclei were assessed, and each isolate was measured three times
Gating strategy	We set the electronic trigger to the FL2 channel (the 580/42 optical filter in front of the 2nd PMT collecting yellow orange emission from the initial 488nm laser line excitation) to focus solely on events that are propidium iodide (PI) positive, i.e. those that contain nucleic acid. We used an appropriate internal sizing control (Oryza sativa nuclei) of known genome size to set the detector voltage on the 580/42 detector to approximately 500V, which enabled identifying small (Bremia) and large (rice) genome sizes simultaneously on scale. No FSC and/or SSC gating was performed as this is unnecessary for simple single fluorescence DNA content flow cytometry measurements.

☐ Tick this box to confirm that a figure exemplifying the gating strategy is provided in the Supplementary Information.
